# The Emerging Role of Interdisciplinarity in Clinical Psychoanalysis

**DOI:** 10.3389/fpsyg.2021.659429

**Published:** 2021-05-05

**Authors:** Dagmar Steinmair, Henriette Löffler-Stastka

**Affiliations:** ^1^Department of Psychoanalysis and Psychotherapy, Medical University of Vienna, Vienna, Austria; ^2^Karl Landsteiner Private University for Health Sciences, Krems an der Donau, Austria

**Keywords:** unconscious, meta-theory, brain, mind, encoding of memory, affective experience

## Abstract

Given the tight interconnections proposed between brain and psyche, psychoanalysis was conceptualized as an interdisciplinary theory right from the beginning. The diversification of knowledge performed by different science and technology fields, concerned with the same matter (explaining mind and brain and connecting them), makes this interdisciplinarity even more visible and evident. This challenges the integrative potential lying in psychoanalytic meta-theory.

## Conceptual Analysis

The scientific discourse is characterized by a constant questioning of truths and requires an openness to innovations and tolerating uncertainty of existing knowledge. When putting distinct strains of knowledge into relation to the whole, the circular character of interpretation of findings is impossible without considering context (hermeneutic circle; Holm-Hadulla, 2003). The psychoanalytic method underlies psychoanalytic therapeutic work as well as psychoanalytic research. It involves “exploration, validation, refutation and discovery”(Bellak, 1961). However, Freud distinguished between gaining (conscious) insights, unconscious drives and conflicts, and psychoanalytic research. Clinical observations are implemented in hypothesis generation, but verifying such theories requires hypothesis testing, according to scientific guidelines, to avoid overgeneralization from individual reflections. When investigating the human mind, psychotherapists have been aware of the overlaps with associated fields (e.g., neuropsychology, neurology, neuroradiology, philosophy, and humanities) and the role psychoanalysis has as a meta-theory. Embedding psychodynamic thinking with an evidence-based approach integrates different perspectives by applying a mixed-method approach, thus doing justice to the investigated matter’s complexity.

When Freud founded the science of the unconscious, psychoanalysis, he did not invent it – literature and philosophy already had shed light on it and described the phenomena emerging from it. The notion of the unconscious (gr. alogia), defining what is not accessible by conscious processing but pushes and clusters around the conscious (gr. logos), was discussed by prominent thinkers like Euripides, Sokrates, Nietzsche, and Schopenhauer (Müller, 2012). Nevertheless, when Freud re-introduced this topic and its applicability to mental disorders, he encountered resistance and controversy. In his view, the debate was propagated due to the humiliation the awareness of unconscious drives evokes. Furthermore, the contraposition psychoanalytic theory proposed against contemporary idealism might have contributed to the resistance (Freud, 1900). With the introduction of a censoring instance determining which unconscious and preconscious contents reach consciousness, the relationship between alogia and logos became a dynamic and potentially influenceable one (Freud, 1900). The threat posed by unconscious and previously mainly uncontrollable contents and drives was enriched by the perspective of a mediating mental function.

Freud (1925) proposed several models when trying to conceptualize the functioning of the human mind, processes, and mental structures. By definition, models are always a simplification of the matter- abstractions of reality, without claiming that they should be exact reproductions of the complex processes they aim to approximate. Thus, also psychoanalytic models changed over time- when different viewpoints had to be integrated.

In general, the effect of psychotherapy has been shown (Leuzinger-Bohleber et al., 2020)-highlighting the importance of common factors for therapy benefit (including: alliance, empathy, expectations, cultural adaptation, and therapist differences, see (Wampold, 2015). Beside the common factors specific factors apply and are necessary for treatment success – treatment differences and specific ingredients as well as adherence and competence decide whether therapists are effective (Wampold, 2015), again resulting in fewer fluctuations across different patients’ therapies. Nevertheless, the formulation of generalizable outcome parameters has repeatedly been questioned and the need of defining outcome in a multidimensional way has been suggested (Diener et al., 2007). Psychotherapy goals are individually different, and thus every outcome parameter applied in research is nothing more than a surrogate. Thus, research in this field is complex and requires investigation of processes and therapeutic interactions during an ongoing therapy process within long-term treatment studies to show improvement not only in surrogate endpoints. However, the high costs of therapy so far need to be paid by the participants themselves over the years, even if a research context applies. This is problematic especially when taking into account that due to the desirable randomized double-blind design of clinical studies patient’s preferences (therapy method, setting, and therapist) would have to be neglected (Leuzinger-Bohleber et al., 2020). When confronting therapy methods and settings with different frequency of treatment, how to exclude dose effects and various mediator variables?

What are the specific factors contributing to therapy success, what makes psychotherapists “effective,” is it possible to establish an interdisciplinary psychotherapists’ competence framework or do they differ across disciplines and therapy schools? Several attempts have been made to operationalize psychoanalytic core competencies and a transparent analysis of the psychoanalytic mindset (Tuckett, 2005; Bush, 2013; Parth and Loeffler-Stastka, 2015). The mechanism of change postulated by Bush (2013) for psychoanalysis relies on the ability and goal of creating “a shift in a patient’s relationship to his mind.” Through providing a sphere of reflection instead of enactment (language, gesture, mimic, etc.), symbolic thinking is enabled (Bush, 2013; Ginsburg, 2016). Psychodynamic treatments re-evoke memories, aiming at a reconsolidation of them through meaningful interactions in the psychoanalytic relationship, thus approaching them differently and provoking alternative associated memory traces (Scully et al., 2017; Leuzinger-Bohleber et al., 2020).

The development of human cognitive abilities and behavior depends on brain development and is experience dependent. Brain plasticity and sensitivity to influences are dependent on age- susceptibility to environmental factors (e.g., stress, sensory and motor experiences, drugs, hormones, and relationships) is higher at a younger age (Giedd et al., 1999; Kolb and Gibb, 2011; Morita et al., 2016). In studying developmental processes Melanie Klein’s clinical work relied on infant and early mother-child relationship observation, trying to infer mental states and the relevance of external and internal factors (Sherwin-White, 2017). However, Klein assumed mental conflicts from the beginning of life caused by innate drives and fantasies and resolved by distorting defense mechanisms. Inspired by Klein’s theories, Stern performed experimental and clinical studies testing the hypothesis that the early object relations are internalized appropriate to reality.

The body of evidence on the importance of therapeutic interventions related to the patient’s affectivity for a positive therapy outcome is growing and investigation of emotion regulation in patients has been identified as a trans-diagnostic factor (Schäfer et al., 2017; Taylor et al., 2020). An intensive experiencing and a modified understanding of the patients’ affects and defense mechanisms are necessary for successful psychodynamic therapies (Diener et al., 2007). Enactments and consciously and unconsciously expressed signals are interpreted and used to draw conclusions about conscious and unconscious mental states. To study the mind and brain connection nowadays psychoanalysts rely on research methods from various associated fields.

Computational and system dynamics modeling of psychoanalytic processes (i.e., nonlinear systems theory) allow for a sophisticated analysis of cognitive processes like dream work, for example. In conceptualizing the human psyche as a self-organizing process revealing dynamic pattern transitions awake as much as while asleep, the dream could just be the way humans get access to more affective-loaded dimensions and meanings. Automated text-analysis and affect charge-analysis together with time-series analysis of dream narratives carried out as process analysis was able to detect the change-point in a psychotherapeutic process (Gennaro et al., 2020).

Topological approaches aiming at formalizing some core aspects of psychoanalytical models rely on neuroimaging studies and neuropsychological concepts.

The detection of the default mode network (DMN) as a functional brain network active when individuals are undisturbed or carry out an undirected mental task (e.g., mind-wandering or daydreaming, thinking about their individual plans/memories) led to attempts to apply this finding to traditional psychodynamic views. DNM is active during mental processes that overlap with and constitute the concept of the Ego (Carhart-Harris and Friston, 2010; Rizzolatti et al., 2014; Lauro-Grotto, 2021). DNM is also active when dreaming. The ventromedial prefrontal cortex (vmPFC) as a part of the DNM regulates motivation, and if damaged dreaming is no longer possible (Zellner, 2013). Thus, the psychoanalytic concept of dreams arising from wishes is supported by findings from imaging and lesion studies. Grotto discusses the concepts of mirroring (pre-reflective, motor simulation; mirror neuron system) and symmetrization (i.e., considering mental processes in relation to external reality; DNM)- theoretical concepts describing embodied cognition.

Mechanisms behind the expression of mimic expressions appearing only for milliseconds (40–500 ms; Ekman and Friesen, 1978) happen at the limit of accuracy of temporal resolution of the applied metabolic based non-invasive functional imaging methods used in human neurosciences (fMRI, PET, NIRS, etc.). The temporal resolution of non-invasive electrophysiological-based (EEG, MEG) methods is higher- with a lower spatial resolution. Burle et al. (2015) investigate how the impossibility to temporally separate different activations with a given imaging technique also degrades the imaging techniques’ spatial resolution and vice versa. The low temporal resolution of metabolic processes may also mask temporally separated activations into a single, more spread one (Burle et al., 2015). Thus, combining the different imaging methods might so far be the best approach in research in humans. All the neuro-imaging methods discussed are not feasible when studying psychotherapeutic interactions in real-time but when investigating brain circuits and networks possibly relevant for mental health, like emotion recognition, memory and theory of mind, this research field is relevant also for psychoanalysts. For Lacan (1959-1960), the concept of memory was not a biological or psychological one – thus highlighting the different semantic levels of the term (Diener et al., 2007). Psychoanalytic work as mentioned above is concerned with memory retrieval and especially with problems in memory retrieval. The symbolic history of the subject is constituted of those contents that together form a signifying chain- with forgetting as an erasure of signifiers (Gennaro et al., 2020). In current neurosciences regarding autobiographical memory a brain network consisting of the prefrontal medial (facilitating retrieval monitoring) and the posteromedial cortical brain regions (enabling the visual imagery processes of memory) has been identified (Thome et al., 2020). Memorizing is thought to be dependent on the arousal and stress level at encoding and on the emotional value the content plays for the individual (Shields et al., 2017). Negative affective linkage together with high arousal leads to a more vivid memory at retrieval and to a tendency to remember the event. A meta-analysis on memory retrieval in post-traumatic stress disorder, interpreted reduced common activation of prefrontal cortices as a bias to re-experiencing with a dysfunction in the possibility to retrieve trauma-associated memories in a controlled manner (Thome et al., 2020). Thus, also current work is preoccupied with the whereabouts when memorizing fails- with Freud (1920) suggesting that when content is overwhelming at encoding, retrieval will happen in a dysfunctional manner. For post-traumatic states a disability to integrate the content in the signifying chain (Lacan, 1955-1956); a differentiation between “traumatic memory” (i.e., unconsciously repeating the past) vs. narrative memory (i.e., narrating the past as past) has been suggested (Dayan and Olliac, 2010). Contemporary conceptions of post-traumatic stress although having their roots in Freudian descriptions of hysteria and dissociation/conversion, assume a deregulation of the stress system (e.g., heightened activation of the amygdala) as an answer to threat-related stimuli (Freud, 1920).

Models associated with message processing have postulated that emotion could be influencing on the depth of information processing (Nabi, 1999). Other theories indicate that emotion influences on the ability of processing information and influence motivation (Chaiken, 1987). Thus, “the extent to which an individual elaborates on a given material” (McKasy, 2020) could be influenced by emotions. Communication scientist McKasy (2020) conducted a meta-analysis to review evidence concerning anger and information processing as compared to a neutral control, sadness, happiness, and fear group. It revealed that no significant influence of such nuanced emotions on information processing was detectable. Even when confronting angry individuals with neutral ones, the meta-analysis found an overall effect size only close to significance – but with an opposite (i.e., positive) effect of anger on information processing (McKasy, 2020).

With the social influence area, the question how information processing is influenced, especially by persuasive communication is very relevant. The persuasive impact of communicator and contextual variables (e.g., expertise, source credibility, attractiveness, liking, interpersonal similarity and message cues like length, number of presented arguments) has also been investigated in the field of cognitive psychology (Chaiken, 1987). The hypothesis that often not much cognitive effort is performed when judging the validity of an argument has been confirmed by empirical evidence, again the role of motivation and ability has been highlighted (Chaiken, 1987).

Neuroimaging studies so far showed substantial overlap in the detection of brain areas relevant for emotion processing (Botvinik-Nezer et al., 2020). However, analysis of the same dataset by different teams performed in the above-mentioned study also showed variability of findings; thus open-data-approaches suggest that the reliability of research relying only on fMRI is questionable.

Multiphoton fluorescence microscopy provides a higher spatial and temporal resolution than the other *in vivo* imaging modalities discussed above; with this method, cellular and subcellular processes can be investigated, and two- and three-dimensional images can be collected with a high temporal resolution (Dunn and Young, 2006). However, to study the human brain, the depth to which this imaging method can be applied is limited.

Already Darwin observed that facial behavior was similar across humans from different ethnic groups and even across species and drew conclusions about evolution of facial communication in mammals (Waller et al., 2020). The Facial Action Coding System (FACS) developed by Ekman and Friesen (1978) based on the work of the anatomist Hjortsjö (1970) is able to measure facial behavior based on individual facial muscle movements, recently Waller and colleagues applied this method to various non-human primate species (Waller et al., 2020). Trained investigators can identify subliminal facial micro-expressions with real-time computed face detection systems (FACS; Ekman and Friesen, 1978). Existing evidence suggests that they represent more than motion artifacts and reflect inner, mostly unconscious emotional states. That facial expression provides means to infer emotion states has lately been investigated in mice (Dolensek et al., 2020). Five stereotyped facial expressions with measurable, completely different features were expressed in mice depending on applied emotionally relevant stimuli. They were interpreted as pleasure, disgust, nausea, pain, and fear; even a range of relative strength was shown depending on stimulus strength and contextual factors.

Thus, facial expressions in mice were not only expressed as a reaction to the trigger (e.g., tasting something sweet or bitter, or when anxiety is provoked) but were dependent on the condition of the individual mouse prior/during the application of the stimulus and context (e.g., saturated vs. hungry). From their experiments, the researchers conclude that facial mice expressions mainly reflect inner emotional states. Due to sufficient similarity – by investigating emotions in the mice-model, research in humans could eventually benefit. Most notably, given that mice brains can be studied *in vivo* with the imaging mentioned above technic (two-photon microscopy), individual insular mice-neurons’ linkages to single emotions were shown (Dolensek et al., 2020).

Advances in informatics and video acquisition technology nowadays allow for a real-time automatic analysis of human expressions (Oh et al., 2018). With the ability to measure even very fast and subtle facial expressions (micro-expressions), recognition of signals expressed voluntarily or unconsciously became possible with FACS. However, the analysis of facial behaviors remains demanding because of its dynamic character and the blend of expressions limiting the description and analysis of these features (Waller et al., 2020). FACS is a flexible, descriptive instrument applicable during social interactions but the interpretation of findings relies on the categories deducible for the observer.

This method was applied to the research on effects of therapeutic interventions partly based on interpretation of body language. Detection and interpretation of subliminal signals unconsciously enacted by the patient through language, gesture, and mimic are relevant when investigating affect expression, regulation, or perception as so far suppressed affective or mental states are made accessible (Diener et al., 2007). With FACS analysis it has been concluded that interpretative and confrontational interventions are associated with contempt expression but result in a better working alliance in psychodynamic treatment (Datz et al., 2019). An initial expression of contempt (e.g., *via* facial micro-expression) could also be interpreted as a signal affect related to the necessary reality check when presented information at first impression does not fit pre-existing assumptions- thus indicating an ability to critically acclaim presented information. Eventually it could be a remnant of impressive or threatening behavior relevant in dealing with the social costs of group living. The subsequent working-through process proposed by psychotherapy is only possible in a secure environment, as hopefully provided within therapeutic relationships. The therapists work relies on the patient’s ability to trust, usually achieved during early development- in highly interpersonal process relying on the ability to relax epistemic vigilance where appropriate, to follow human’s instinct for social learning and adaptation to a social environment that can be trusted as a reliable source of information (Fonagy and Campbell, 2021).

Prediction of psychic functioning in adulthood from childhood or adolescent psychic symptoms or disease is not possible. A cultural-developmental model of vulnerability to mental disease has recently been proposed together with the hypothesis that there might be a “general factor that underpins an individual’s level of vulnerability,” relevant for vulnerability to mental diseases in general (Fonagy and Campbell, 2021). However, common mental disorders are frequent in adolescents, a recent meta-analysis showed a prevalence of 25% globally (Silva et al., 2020). More than 50% of mental disorders begin at the age of <14 years old and 75% at <25 (Kessler et al., 2007). Therefore, preventive approaches in the mental health sector now focus on youth research, identifying prediction parameters for a favorable outcome and specific therapy factors leading to improvement. So far, the evidence points to the importance of early interventions of the social environment for mentalizing and affecting regulation abilities necessary for social cognition and overall mental functioning (Correll et al., 2018; Sandbank et al., 2020; Fonagy and Campbell, 2021; Luyten et al., 2021). However, long-term RCTs of applied methods are not jet available- but poor outcomes in adults with psychic conditions rooting in childhood and adolescence demand new approaches.

As mentioned above, existing evidence suggests that unconscious but detectable signals are enacted due to internal emotional states and reactions to relevant triggers applied by the environment. Socially transmitted interactional patterns are actualized in any given social encounter- they unfold their impact through the preconscious and unconscious enactments (Balint, 1957). If mentalizing abilities are compromised (transiently due to current states or permanently due to traits), an adaptation to the situation at hand remains impossible- thus, patterns might get perpetuated rather than enriched by social learning. To break this inflexible enactment, therapeutic interventions focusing on the affective interplay in the therapeutic relationship aim at providing the conditions for a reshaping and reframing of this interplay- facilitating learning through (repeated) interaction (Leuzinger-Bohleber et al., 2020).

The psychoanalytic theory assumes that we would undervalue humans’ cognitive functions by focusing only on consciously available knowledge. Most of the expressed human thoughts, affective and emotional states, and more complex behavior (like social interactions) would be unexplainable. Thus, implicitly stored knowledge, “embodied knowledge has been proposed: that is, information that is uniquely and integrally embodied in the person’s personality, creativity, intelligence, perceptions, experiences, and relationships. Embodied knowledge is the essence of expertise” (Fitzpatrick, 2003).

When investigating psychotherapeutic competence, such embodied knowledge would apply. Embodied learning is achieved through lived experience; its verbalization and transmission are limited (Stern, 2007).

The importance of affective linkage of long-term memory contents might root in the apparent advantage of remembering information loaded with subjective meaning. Editing memories together with associated emotional memory traces seems to be possible at several time points: when encoding and again at retrieval (Schlichting and Preston, 2015; Scully et al., 2017; Shields et al., 2017). Mental health is associated with a bias toward remembering more events with an affectively favorable loading- or even reshaping memories toward a more pleasurable memory, aiming at an optimistic narrative (Muir et al., 2015, 2017; Phelps and Hofmann, 2019). Implicit bias is almost inevitable, as abstraction and creating somewhat stereotypical patterns from seemingly similar experiences might offer advantages, at least when fast reactions are necessary. Psychoanalysis seeks to strengthen the ability to cope with given memory contents and an adequate reality check and analysis of associated traces at the actualization, enriching them with new associable hints generated through the newly acquired implicit relational knowledge experienced in the therapeutic relationship.

Psychoanalytic therapy nowadays, should be part of a patient-oriented interdisciplinary network involving case-management (social-work, general practitioner) and when needed psychiatrist (incl. prescribing of medicine) and other relevant consultants (e.g., neurologist, internist, and gynecologist), laboratory and imaging. The importance of involvement of the patient’ social network and parties affected and if not applicable the establishment of a peer group (e.g., through group therapy) has been shown (Renner et al., 2013). Mental health centers would certainly save time and resources by facilitating collaboration and communication especially when dealing with severely ill mental disease, as relapse rates are high and secondary complications and consequences often result in limited ambulatory possibilities.

For research, to conclude, when aiming at the generation of common ground with associated fields [e.g., neurosciences, (cognitive) psychology, philosophy, pharmacology, anatomy, (neuro-)biology, social sciences, humanities, and communication sciences], the inherent bias remains the same. When investigating the mind-brain connection, the object of study explores itself. However, integrating different viewpoints and analysis with various methods should lead to a more complete image acquired by interdisciplinary communication and collaboration rather than fragmentation of resources. Investigating mechanisms of change relevant to clinical psychotherapeutic work remains challenging.

## Author Contributions

HL-S conceived the conceptual work and specified the research fields from her clinical and research experience. DS wrote and discussed iteratively the main domains with HL-S. Both the authors contributed to the article and approved the submitted version.

### Conflict of Interest

The authors declare that the research was conducted in the absence of any commercial or financial relationships that could be construed as a potential conflict of interest.
